# Protective Effect of Decursin Extracted from *Angelica gigas* in Male Infertility via Nrf2/HO-1 Signaling Pathway

**DOI:** 10.1155/2016/5901098

**Published:** 2016-02-16

**Authors:** Woong Jin Bae, U. Syn Ha, Jin Bong Choi, Kang Sup Kim, Su Jin Kim, Hyuk Jin Cho, Sung Hoo Hong, Ji Youl Lee, Zhiping Wang, Sung Yeoun Hwang, Sae Woong Kim

**Affiliations:** ^1^Catholic Integrative Medicine Research Institute, College of Medicine, The Catholic University of Korea, Seoul 137-701, Republic of Korea; ^2^Department of Urology, College of Medicine, The Catholic University of Korea, Banpo-daero 222, Seocho-gu, Seoul 137-701, Republic of Korea; ^3^Department of Urology, Second Hospital of Lanzhou University, Lanzhou, China; ^4^Korea Bio Medical Science Institute, Seoul, Republic of Korea

## Abstract

Higher testicular temperature results in altered spermatogenesis due to heat-related oxidative stress. We examined the effects of decursin extracted from* Angelica gigas* Nakai on antioxidant activity* in vitro* and in a cryptorchidism-induced infertility rat model. TM3 Leydig cell viability was measured based on oxidative stress according to treatment. Either distilled water or AG 400 mg/kg of* A. gigas* extract was administered orally for 4 weeks after unilateral cryptorchidism was induced. After 1, 2, and 4 weeks, six rats from the control group and six rats from treatment group were sacrificed. Testicular weight, semen quality, antioxidant activities, nuclear factor erythroid 2-related factor 2 (Nrf2) protein, and mRNA expression of Nrf2-regulated genes were analyzed. Treatment with* A. gigas* extract (1) protected TM3 cells against oxidative stress in a dose-dependent manner, (2) improved the mean weight of the cryptorchid testis, (3) maintained sperm counts, motility, and spermatogenic cell density, (4) decreased levels of 8-hydroxy-2-deoxyguanosine (8-OHdG) and increased levels of superoxide dismutase (SOD), (5) significantly increased Nrf2 and heme oxygenase-1 (HO-1), and (6) significantly decreased apoptosis. This study suggests that decursin extracted from* A. gigas* is a supplemental agent that can reduce oxidative stress by Nrf2-mediated upregulation of HO-1 in rat experimentally induced unilateral cryptorchidism and may improve cryptorchidism-induced infertility.

## 1. Introduction

Infertility, which can be explained as the failure of a couple in trying to conceive after one year of frequent, unprotected sexual intercourse, is a serious clinical issue that affects 13–15% of couples worldwide [1]. Male infertility is responsible for 60% of cases involving conceptive couples with pregnancy-related problems [2]. Sperm is produced by the highly complicated process of spermatogenesis, and partial or complete interruption of spermatogenesis ultimately leads to oligospermia or azoospermia.

Observation study on male infertility with oligozoospermia or azoospermia, in particular, suggests that some patients may have testicular heat exposure due to an intrinsic defect in scrotal thermoregulation, varicocele, or work hazard [3]. Several studies report that testicular hyperthermia above normal ranges causes impaired spermatogenesis due to heat-related oxidative stress on the seminiferous tubules [4, 5]. Moreover, nuclear factor erythroid 2-related factor 2 (Nrf2) plays a significant role in preventing the development of oxidative stress in spermatogenesis [6]. Yu et al. [7] also demonstrated a strong correlation between functional discrepancy in Nrf2 promoter gene and abnormal spermatogenesis in humans.

Decursin, a major active ingredient from* Angelica gigas* Nakai (Apiaceae), has been reported to inhibit the growth of various cancer cells through cell cycle arrest and apoptosis [8, 9]. In addition, a protective effect of decursin has been suggested against the neurotoxicity in animal cortical cells [10]. Even further, decursin plays a major role as free radical scavenger and activated the upregulation of heme oxygenase-1 (HO-1) expression through stimulation of Nrf2, conferring protection against oxidative damage in rat pheochromocytoma (PC12) cells [11].

We examined the effects of decursin extracted from* Angelica gigas* on antioxidant activity* in vitro* and in a cryptorchidism-induced infertility rat model. We hypothesized that decursin-induced HO-1 expression would protect against heat stress-induced degeneration of testicular germ cells and apoptosis.

## 2. Methods

### 2.1. Preparation of* Angelica gigas* Extract and Characterization of Decursin

**Figure 1:**
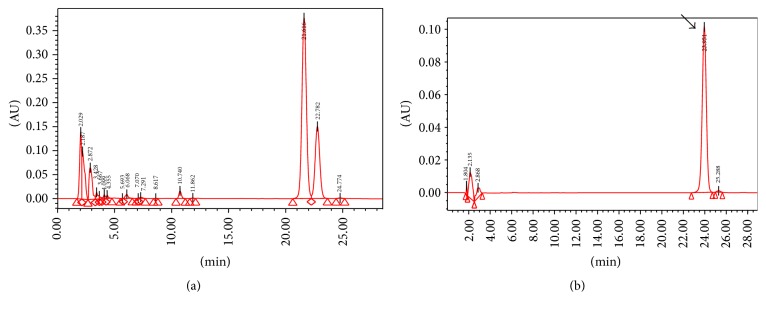
HPLC chromatogram of the extract of* Angelica gigas* (a) and the standard solution (b). The peak with the arrow indicates decursin in standard compounds. A corresponding peak was seen in the extract HPLC chromatogram.

The extract of* Angelica gigas* used in our study was produced using the following method: commercial* Angelica gigas* roots were extracted with 12,000 mL of 30% ethanol for 3 hours at 90–100°C. The extracts were filtered twice through a 50 *μ*m and a 1 *μ*m filter and concentrated in vacuo and then lyophilized. Decursin in the* Angelica gigas* extract was analyzed and quantified by high performance liquid chromatography (HPLC) using Waters 2695 Preparation Module HPLC system (Waters Corporation, MA, USA). Several peaks were obtained in the HPLC chromatogram by diode array detection (DAD) at 230 nm. The major peak was identified as decursin by comparison with the standard compound (Figure 1). As a result of this assay, decursin content was quantified as 37.6 ± 2.2 mg/g.

### 2.2. Cell Viability Test* In Vitro*

TM3 cells, an immature mouse Leydig cell line (Korean Cell Line Bank, Seoul, Korea), were cultured in Dulbecco's modified Eagle's medium (DMEM)/F-12 medium (GIBCO, Life Technologies Co., USA) supplemented with 10% heat-inactivated fetal bovine serum (FBS; GIBCO) at 37°C. Cells (75,000 cells/mL medium/well) were plated in 24-well cluster dishes, unless otherwise specified. They were seeded on 96-well plates in 10% FBS/DMEM/F-12 and breeded for 24 hours. They were pretreated with the* Angelica gigas* extract for two hours and treated with 40 uM hydrogen peroxide (H_2_O_2_) for two hours to create oxidative cellular stress. Afterwards, alamarBlue (Invitrogen, USA) was added to the cells, and the intensity of the presented color was measured at 570 nm using ELISA Reader (Molecular Devices, USA) after incubating for 3 hours. Cell viability was calculated as previously described [12].

### 2.3. Animal Groups and Treatment Protocol

This study was investigated in strict accordance with the recommendations in the Guide for the Care and Use of Laboratory Animals of the National Institutes of Health. The protocol was approved by the Institutional Animal Care and Use Committee in School of Medicine, The Catholic University of Korea (CUMC-2012-0168-01). Thirty-six 8-week-old male Sprague-Dawley rats were treated under an approved protocol. Unilateral cryptorchidism in rats was surgically induced as previously described [13]. Animals were anesthetized, and a midline abdominal incision was performed. The gubernaculum on the left side was cut to displace the testis into the abdomen, and the inguinal canal was closed. The contralateral testis was sham-operated to act as the paired control; the gubernaculum was cut and the inguinal canal reconstructed. Either distilled water (*n* = 18, control groups) or 400 mg/kg of* Angelica gigas* extract (*n* = 18, treatment groups) was administered orally for 4 weeks after unilateral cryptorchidism was induced.* Angelica gigas* extract was dissolved in distilled water and administrated orally once a day. After 1, 2, and 4 weeks, six rats from the control group and six rats from treatment group were sacrificed and blood samples were collected. The testes and epididymides were resected after anesthesia and measured.

### 2.4. Evaluation of Cauda Epididymal Sperm Count and Motility

Samples of spermatozoa were collected from the caudal region of epididymis by mincing it finely in normal saline containing 0.5% bovine serum albumin at 37°C and then were filtered. Sperm suspensions were analyzed as previously described [14]. The sperm count represents the number of sperms in 1 mL of the medium. Sperm motility is expressed as the percentage of sperm that showed any movement.

### 2.5. Measurement of Spermatogenic Cell Density

Testicular tissues procured were fixed, embedded in paraffin, stained with haematoxylin-eosin, and inspected under a light microscope at ×400 magnification. Ten characteristic sites in seminiferous tubules were selected randomly and spermatogenic cell density was measured. It was calculated as the diameter of germinal cell layer divided by the width of the seminiferous tubule [15].

### 2.6. Measurement of Oxidative Stress

Oxidative stress was assessed by measuring the 8-hydroxy-2-deoxyguanosine (8-OHdG) content and superoxide dismutase (SOD) activity quantitatively. Total DNA was extracted from the testis using the DNeasy Blood & Tissue kit (Qiagen, Valencia, CA, USA). The level of 8-OHdG was measured with a DNA oxidation kit (Highly Sensitive 8-OHdG Check ELISA; Japan Institute for the Control of Aging, Fukuroi, Japan). After the final color was developed with the addition of 3,3′,5,5′-tetramethylbenzidine, absorbance was measured at 450 nm. Tissue sample concentration was measured from a standard curve and corrected for DNA concentration. SOD activity (CuZnSOD and Mn SOD) in tissue was determined using SOD Assay Kit-WST (Dojindo) and the decrease in the rate of superoxide-mediated reduction of nitroblue tetrazolium monitored at 450 nm using a spectrophotometer.

### 2.7. Western Blot Analysis

Western blot was performed by the standard method. Equal amounts of proteins were fractionated by SDS-PAGE gel electrophoresis and electrotransferred to Immun-Blot PVDF membrane (0.2 *μ*M pore size, Bio-Rad). Membranes were blocked overnight at 4°C in Tris-buffered saline (TBS), 0.05% (v/v) Tween-20, 150 mM NaCl, and 5% (w/v) bovine serum albumin (BSA, Santa Cruz Biotechnology, Santa Cruz, CA, USA), followed by 2 hours of incubation with primary antibody diluted in the same buffer. Immunoblot analysis was carried out using anti-Nrf2 (1/250), anti-HO-1 (1/1000), anti-Bax (1/1000), and anti-Bcl-2 (1 : 1000) polyclonal antibody (Abcam Co., UK). After washing with TBS-T (TBS, 0.1% Tween 20), the membrane was incubated with anti-rabbit IgG AP-linked secondary antibody and then washed with the same buffer. The immunoblotted membrane was developed with BCIP/NBT color-developing solution. The blots in the samples were quantified by densitometry analysis using PDQuest software (Version 7.0, Bio-Rad, Hercules, CA, USA).

### 2.8. Quantitative Real-Time PCR

The frozen testes were homogenized with TRIzol reagent (Invitrogen, Carlsbad, CA, US) to extract mRNA and cDNA synthesis was performed using the SuperScript 3 First-Strand kit (Invitrogen, Carlsbad, CA, US) according to the manufacturer's information. Gene-specific primers were determined based on the corresponding mRNA sequences with Primer Version 5.0 (Table 1). PCR amplification of cDNA was performed in a real-time PCR machine step on plus (Applied Biosystems) with SYBR Green PCR Master Mix (Invitrogen) as indicated: 2 minutes at 50°C for dUTP activation and 10 minutes at 95°C for initial denaturation of cDNA followed by 40 cycles, each consisting of 15 s of denaturation at 95°C and 60 s at 60°C for primer annealing and chain extension.

### 2.9. Statistical Analysis

Statistical analyses were performed using SPSS 16.0 (SPSS Inc., Chicago, USA). The data was expressed as mean ± standard deviation. Statistical significance was analyzed by ANOVA test and *p* < 0.05 was considered to be significant.

## 3. Results

### 3.1. Protective Effect against Oxidative Stress in TM3 Cells

**Figure 2:**
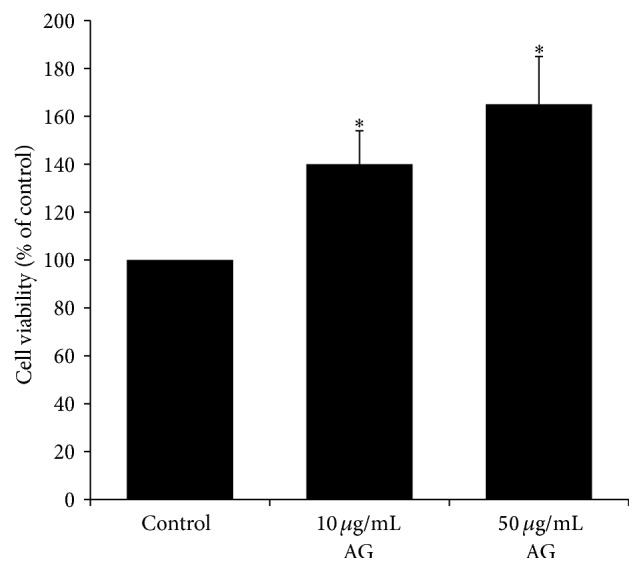
Protective effects of* Angelica gigas* extract against H_2_O_2_. The cells were pretreated with the extract of* Angelica gigas* 2 hours before H_2_O_2_ treatment. ^*∗*^ *p* < 0.05 as compared with the control group.

To evaluate whether* Angelica gigas* extract could protect TM3 cells against H_2_O_2_-induced damage, alamarBlue assay was performed. Incubation with H_2_O_2_ significantly decreased cell viability by 40% compared to untreated cells. Increased cell viability was found in a dose-dependent manner (Figure 2). The viabilities were increased to 140% and 165% by treatment with* Angelica gigas* extract concentrations of 10 *μ*g/mL and 50 *μ*g/mL, respectively.

### 3.2. Body and Testes Weights

There was no significant difference in contralateral testicular weights for four weeks. Upon cryptorchidism induction in the first week, there were also no significant differences in left testicular weights between the control group and the treatment group. However, the mean weight of the left testes from the treatment group was significantly larger compared with the control group on the second and fourth weeks (*p* < 0.05). The mean weights of the left testes are listed in Table 2.

### 3.3. Sperm Counts and Motility

Mean sperm counts and the percentage of motile spermatozoa in the left epididymis are shown in Table 2. There were no significant differences between the control and treatment groups in the first week, but mean sperm counts and the percentage of motile spermatozoa from the control group were significantly lower than those in the treatment group by the second and fourth weeks (*p* < 0.05).

### 3.4. Spermatogenic Cell Density

**Figure 3:**
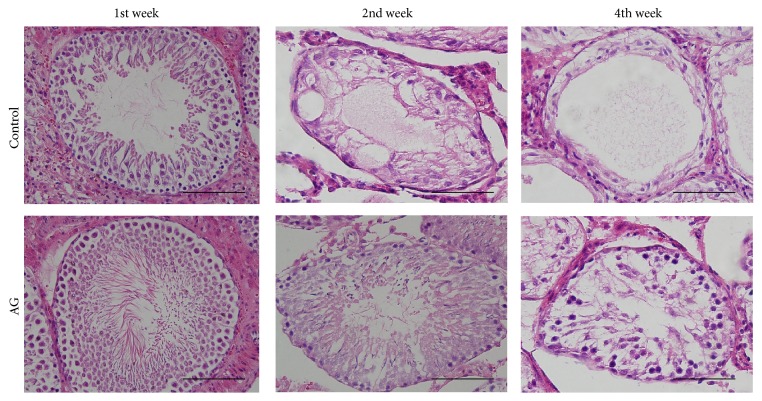
Time-dependent histopathological findings of left testis (haematoxylin and eosin stain). Scale bars shown in each figure represent 100 *μ*m. AG, the extract of* Angelica gigas* treatment.

Upon cryptorchidism induction in the first week, considerable spermatocytes lined up the germinal cell layer in the control and the treatment groups (Figure 3). On the other hand, the seminiferous tubule is shrunken and the germinal cell layers are decreased in the control group after 2 weeks. The germinal cell layer in the treatment group was thicker compared with that of the control group by the second and fourth weeks (Table 2, *p* < 0.05). There was no significant difference in contralateral testicular spermatogenic cell density.

### 3.5. Measurement of Oxidative Stress

**Figure 4:**
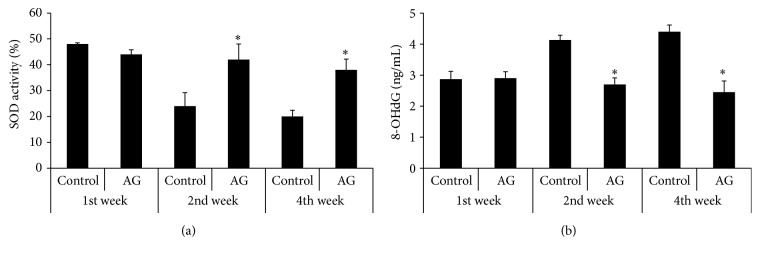
Comparison of the expression levels of SOD (a) and 8-OHdG (b). AG, the extract of* Angelica gigas* treatment. ^*∗*^ *p* < 0.05 as compared with the control group at the same period of time.

Mean expression of oxidative stress markers in the left testes is shown in Figure 4. There were no significant differences in 8-OHdG and SOD expression between the two groups in the first week. A time-dependent increase in 8-OHdG and a decrease in SOD were examined in the control group, but oxidative stress was observed to be significantly lower in the treatment group at the same period of time (*p* < 0.05).

### 3.6. Expression of Nrf2 and HO-1

**Figure 5:**
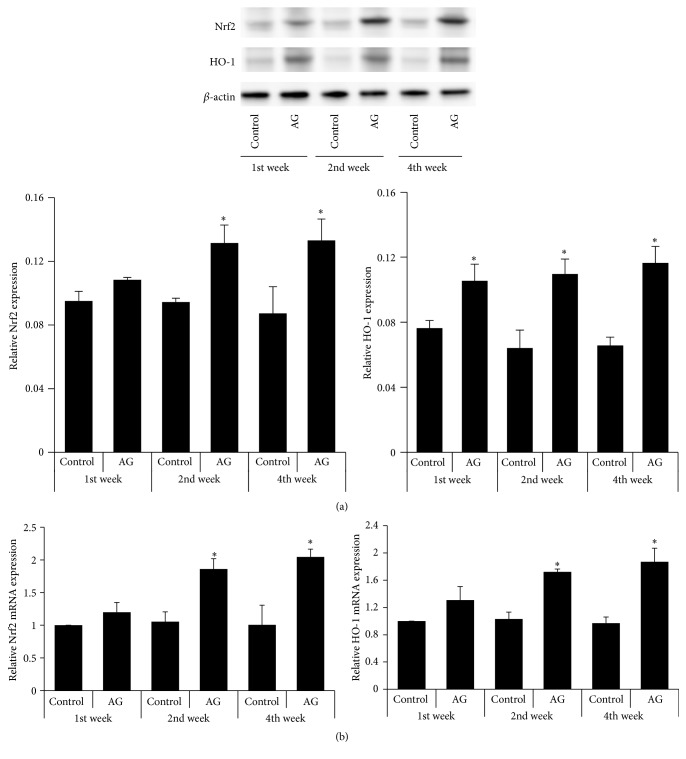
Effect of* Angelica gigas* extract on protein expression (a) and mRNA expression (b) of Nrf2-regulated genes. AG, the extract of* Angelica gigas* treatment; HO-1, heme oxygenase-1; Nrf2, nuclear factor erythroid 2-related factor 2. ^*∗*^ *p* < 0.05 as compared with the control group at the same period of time.

We found that significant increases of Nrf2 and HO-1 proteins were exhibited in the treatment group compared with the control group by the second and fourth weeks (Figure 5(a)). There were no significant differences in Nrf2 and HO-1 mRNA in the control group across time points, but mRNA transcript levels were significantly higher in the treatment group compared with the control group by the second and fourth weeks (Figure 5(b)). Moreover, the progressive increase in Nrf2 levels was firmly associated with an increase in HO-1 expression.

### 3.7. Apoptosis-Related Protein Expression

**Figure 6:**
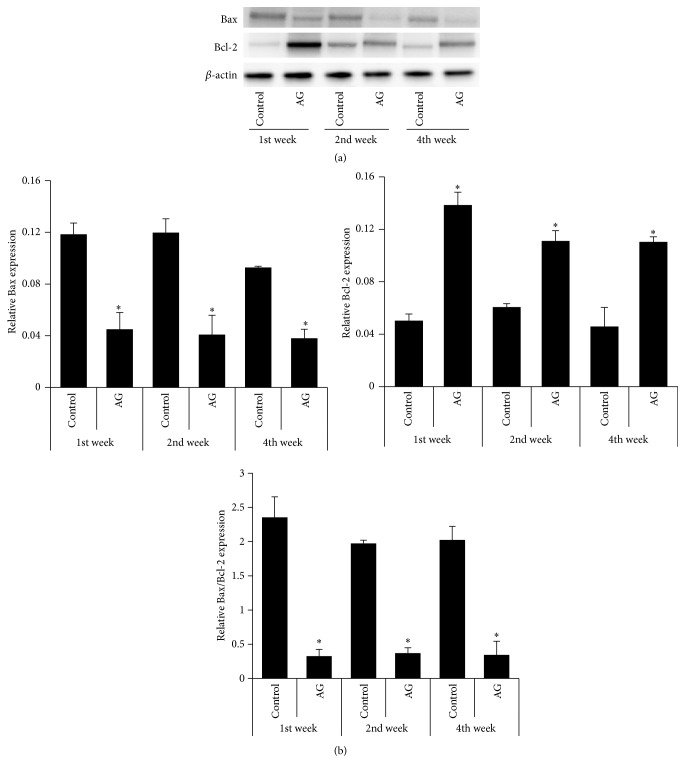
Comparison of the expression levels of Bax and Bcl-2 expression in the testicular tissue. (a) Western blot analysis of Bax and Bcl-2. (b) Densitometric analysis of Bax, Bcl-2, and Bax/Bcl-2 ratio relative to beta-actin. AG, the extract of* Angelica gigas* treatment. ^*∗*^ *p* < 0.05 as compared with the control group at the same period of time.

The expression of proapoptotic (Bax) and antiapoptotic (Bcl-2) proteins was investigated. Treatment with* Angelica gigas* extract significantly declined the level of Bax protein with a collateral increase in Bcl-2 protein compared with the control group over the whole period (Figure 6(a)). These changes decreased the Bax/Bcl-2 ratio, which is considered to be an index to evaluate apoptosis (Figure 6(b)).

## 4. Discussion

The main findings of our study were as follows. The treatment with* Angelica gigas* extract (1) protected TM3 cells against H_2_O_2_-induced oxidative stress in a dose-dependent manner, (2) improved the mean weight of the cryptorchid testis, (3) maintained sperm counts, motility, and spermatogenic cell density, (4) decreased levels of 8-OHdG and increased levels of SOD, demonstrating its antioxidant effect, (5) significantly increased Nrf2 and HO-1, and (6) significantly decreased apoptosis.

We evaluated the protective effect of* Angelica gigas* extract against H_2_O_2_-induced oxidative stress in TM3 cells. In addition, we demonstrated that the mean weight of the cryptorchid testis in the control group was significantly decreased compared with that of the treatment group by the second and fourth weeks. Most mammal testes are more sensitive to heat than other organs, and scrotal temperature is lower than core body temperature [16, 17]. Elevated intratesticular temperature induces oxidative stress, resulting in apoptosis and impairment of spermatogenesis [18, 19]. It is correlated with a decrease in cellular antioxidant defenses or an increase in the production of reactive oxygen species (ROS) [20]. Several studies demonstrated that increased ROS impaired the physiological processes such as capacitation, hyperactivation, acrosome reactions, and signaling processes to provide appropriate fertilization [21]. Thus, antioxidant supplement may help improve the imbalance of an excessive ROS and restore sperm parameters.

Oral supplements and herbal medicines have been proposed to recover male factor infertility [22]. These supplements were reported to improve sperm quality, and their antioxidants are thought to decrease ROS in oligoasthenospermia patients [23–26]. We found that treatment with* Angelica gigas* extract decreases oxidative stress in cryptorchidism-induced rats and posit that the curative effects may contribute to suppression of ROS production. In addition, we examined the detailed mechanism of the antioxidant activity in* Angelica gigas* extract.

HO-1 is a stress-responsive protein shortly activated by variable noxious stimuli as well as oxidative stress. HO-1 has been known as the cytoprotective effect resistant to oxidative stress [27]. The Nrf2 antioxidant system also has been recognized as an important therapeutic target against oxidative stress, producing expression of cytoprotective enzymes and related proteins [28]. Previous studies have reported that oxdative stress resulted in lower epididymal sperm motility in Nrf2 knockout mice [6]. Nrf2 is released and transmits the stress signal to the nucleus for activation of a specific set of genes encoding phase II antioxidant enzymes as well as stress responsive proteins such as HO-1 [29].

Li et al. [30] studied about the oxidative damage on male reproductive organ. The whole body heat stress upregulated Nrf2 expression for about a week, but the significant increased oxidative stress was identified thereafter. Several supplements (e.g., sulforaphane, curcumin, and caffeic acid phenethyl ester) activate the Nrf2 antioxidant response element (ARE) system [31, 32]. Li et al. [11] reported that decursin treatment leads to the upregulation of Nrf2/HO-1 expression in PC12 cells. Decursin may have the possibilities to prevent chemotherapy-induced cytotoxicity via the activation of antioxidant enzyme. We found that decursin extracted from* Angelica gigas* increased Nrf2 and HO-1 in the cryptorchid testis. Moreover, a prevention of decrease in SOD was observed in the treatment group. Therefore, our results suggest that antioxidant defense is reinforced by decursin via activation of Nrf2 and upregulation of antioxidant enzyme activity.

The Bcl-2 family and related proteins are key regulators of apoptosis [33]. They can be classified into two groups: Bcl-2, an antiapoptotic protein, and Bax, a proapoptotic protein [34]. Knudson et al. [35] demonstrated that the Bcl-2 family mainly plays a role during spermatogenesis in Bax-deficient mice. Several studies have reported the importance of the Bcl-2 family in heat-induced oxidative stress on male reproductive function [36, 37]. They suggested the possible involvement of Bcl-2 and Bax in the germ cell apoptosis. We also investigated the germ cell apoptosis induced by cryptorchidism and the antiapoptotic effect of decursin extracted from* Angelica gigas*.

We examined the role of decursin extracted from* Angelica gigas* as a supplemental agent to prevent heat-induced oxidative stress in cryptorchidism. Our limitation is that the more accurate infertility model due to oxidative stress is needed. More defense mechanisms such as Nrf2 and related antioxidative enzyme are required if oxidative stress causes impaired semen parameters, DNA damage, and apoptosis. Antioxidant supplementation as well as lifestyle change like no smoking or balanced diet can be helpful for the natural balance between ROS and antioxidant. Therefore our study may support appropriate evidence for the use of a new complementary and alternative medicine for treating male infertility. In addition, future work should investigate a detailed mechanism of decursin with or without Nrf2 related enzyme activity.

The present study suggests that decursin extracted from* Angelica gigas* is a supplemental agent that can reduce oxidative stress by Nrf2-mediated upregulation of HO-1 and may improve cryptorchidism-induced infertility.

## Figures and Tables

**Table 1:** Primers used for real-time PCR.

Gene	GenBank accession number	Sequence (5′→3′)	Length of DNA product (bp)
Beta-actin (ACTB)	NM_007393	F: CTGTCCCTGTATGCCTCTG	218
		R: ATGTCACGCACGATTTCC	

Nuclear factor erythroid 2-related factor 2	NM_010902	F: CAGTGCTCCTATGCGTGAA	109
		R: GCGGCTTGAATGTTTGTC	

Heme oxygenase-1	NM_010442	F: ACAGATGGCGTCACTTCG	128
		R: TGAGGACCCACTGGAGGA	

**Table 2:** Comparisons of parameters of the cryptorchid testicular health.

		Left testicular weight (g)	Sperm count (×10^6^/g cauda)	% of motile spermatozoa	Spermatogenic cell density
1st week	Control	1.526 ± 0.225	350.2 ± 10.9	20.7 ± 3.3	0.343 ± 0.016
	AG	1.612 ± 0.033	360.6 ± 14.0	22.5 ± 5.2	0.305 ± 0.031

2nd week	Control	1.232 ± 0.165	240.2 ± 13.6	7.6 ± 2.3	0.275 ± 0.043
	AG	1.515 ± 0.367^*∗*^	300.8 ± 12.5^*∗*^	14.5 ± 2.7^*∗*^	0.314 ± 0.074^*∗*^

4th week	Control	0.858 ± 0.285	75.2 ± 18.2	5.7 ± 3.1	0.154 ± 0.028
	AG	1.433 ± 0.634^*∗*^	310.5 ± 14.7^*∗*^	13.2 ± 8.2^*∗*^	0.269 ± 0.052^*∗*^

Data show the mean ± SD AG, the extract of *Angelica gigas* treatment.

^*∗*^ *p* < 0.05 as compared with the control group at the same period of time.
